# Commentary: An artificial intelligence prediction model outperforms conventional guidelines in predicting lymph node metastasis of T1 colorectal cancer

**DOI:** 10.3389/fonc.2024.1337576

**Published:** 2024-02-09

**Authors:** Katsuro Ichimasa, Shin-ei Kudo, Khay Guan Yeoh

**Affiliations:** ^1^ Digestive Disease Center, Showa University Northern Yokohama Hospital, Yokohama, Japan; ^2^ Yong Loo Lin School of Medicine, National University of Singapore, Singapore, Singapore; ^3^ Department of Gastroenterology and Hepatology, National University Hospital, Singapore, Singapore; ^4^ Department of Medicine, National University Hospital, Singapore, Singapore

**Keywords:** T1 colorectal cancer, lymph node metastasis, artificial intelligence, risk factor, variable selection algorithm

## Introduction

Approximately 10% of T1 colorectal cancers (CRCs) harbor lymph node metastasis (LNM), necessitating surgical resection with lymph node dissection for curative intent (1). Accurate preoperative assessment of LNM risk following endoscopic treatment of T1 CRC is critical to determine the need for additional bowel resection. Present guidelines stipulate risk factors for LNM and indications for subsequent surgery post-endoscopic resection, including submucosal invasion depth ≥1000 µm, positive lymphovascular invasion, poor differentiation adenocarcinoma, signet-ring cell carcinoma, mucinous carcinoma, and tumor budding grade (grade 2 or 3) (2). Nevertheless, the current guidelines yield approximately 10% accuracy for LNM prediction, highlighting the necessity for a more precise model to prevent excessive treatment. Although various prediction models for LNM have been proposed, a consensus on the essential predictors and their number for effective clinical application remains a subject for discussion (3–6).

## Comment on the findings and discussion

Piao et al. crafted an artificial intelligence (AI) model employing a classification and regression tree algorithm that outperformed both the Japanese Society for Cancer of the Colon and Rectum in Japan (AUC=0.588) and National Comprehensive Cancer Network guidelines in the US (AUC=0.850), achieving superior discriminative ability (AUC=0.960) (7). This model incorporated a comprehensive array of 12 clinicopathological variables: patient age and sex, tumor size, location, and morphology, histologic differentiation, lymphovascular invasion, tumor budding, poorly differentiated clusters, and the width, depth, and area of submucosal invasion. Their model incorporated a larger number of variables compared to previous models (**Table 1**).

**Table 1:** Variable selection of the predicting model for lymph node metastasis in T1 colorectal cancer.

Author (year)	Design, Country	N, Total	Predicting tool	AUC or C-index	Variables (in order of importance if available)
Pial et al. (7) (this study)	Single center-retrospective, China	651	CART	0.960	12 variables: age, tumor size, area of submucosal invasion, depth of submucosal invasion, lymphovascular invasion, location, morphology, sex, width of submucosal invasion, histological differentiation, tumor budding, poorly differentiated cluster
Kajiwara et al. (6)	Multicenter-retrospective, Japan	4,673	Nomogram	0.790	6 variables: submucosal invasion depth, tumor grade, lymphovascular invasion, location, tumor budding, sex
Kudo et al. (5)	Multicenter-retrospective, Japan	4,073	ANN	0.83	8 variables: lymphatic invasion, vascular invasion, histological grade, morphology, tumor location, sex, tumor size, age
Ahn et al. (4)	SEER data base retrospective, US	43,542	RF	0.991	7 variables: tumor size, age, tumor grade, race, tumor type, primary site, sex
Oh et al. (3)	Multicenter-retrospective, Korea	1,555	Nomogram	0.771	5 variables: vascular invasion, histologic grade, submucosal invasion, background adenoma, tumor budding

AUC, area under the curve; C-index, concordance index; CART, classification and regression tree; ANN, artificial neural network; SEER, Surveillance, Epidemiology, and End Results; RF, random forest.

The selection of variables is crucial to enhance the model’s predictive accuracy and to circumvent overfitting. Three core considerations are paramount in this domain. Firstly, the correlation between the variables and LNM is essential. Along with the established pathological risk factors, recent extensive Japanese multicenter studies have identified a correlation between LNM and variables such as patient age and sex, as well as tumor location and size (5, 6). Notably, Piao’s study highlighted the significant contribution of patient age and tumor size to LNM risk. Secondly, the generality of the information is vital; it must be readily available at any medical facility. While patient age and sex, tumor location and size, along with guideline-specified pathological risk factors, are commonly accessible, the width and area of submucosal invasion, though critical to their AI model, may not be universally available. Lastly, the predictive model’s reproducibility warrants attention. Pathological variables are subject to inter-pathologist variability, unlike stable clinical variables such as age and sex. The interobserver reliability for depth of submucosal invasion, lymphovascular invasion, differentiation, and tumor budding is reported to be relatively low, with kappa values ranging between 0.10 and 0.68 (1). In addition, pathological diagnostic criteria can vary between countries, and the application of immunostaining may differ among institutions.

Piao’s investigation illustrates that an AI-assisted predictive model, integrating clinical with pathological factors, surpasses the accuracy of the current guidelines. The impending challenge lies in discerning the appropriate factors for inclusion and ensuring reproducibility in routine clinical practice.

## Author contributions

KI: Conceptualization, Writing – original draft. S-EK: Writing – review & editing. KY: Writing – review & editing.
